# Identifying Risk Factors Associated with Inappropriate Use of Acid Suppressive Therapy at a Community Hospital

**DOI:** 10.1155/2016/1973086

**Published:** 2016-10-12

**Authors:** Amandeep Singh, Vijay Bodukam, Kirit Saigal, Jaya Bahl, Yvette Wang, Alexandra Hanlon, Yinghui Lu, Michael Davis

**Affiliations:** Crozer Chester Medical Center, Upland, Philadelphia, PA, USA

## Abstract

*Purpose*. By examining the prescribing patterns and inappropriate use of acid suppressive therapy (AST) during hospitalization and at discharge we sought to identify the risk factors associated with such practices.* Methods*. In this retrospective observational study, inpatient records were reviewed from January 2011 to December 2013. Treatment with AST was considered appropriate if the patient had a known specific indication or met criteria for stress ulcer prophylaxis.* Results*. In 2011, out of 58 patients who were on AST on admission, 32 were newly started on it and 23 (72%) were inappropriate cases. In 2012, out of 97 patients on AST, 61 were newly started on it and 51 (84%) were inappropriate cases. In 2013, 99 patients were on AST, of which 48 were newly started on it and 36 (75%) were inappropriate cases. 19% of the patients inappropriately started on AST were discharged on it in three years. Younger age, female sex, and 1 or more handoffs between services were significantly associated with increased risk of inappropriate AST.* Conclusion*. Our findings reflect inappropriate prescription of AST which leads to increase in costs of care and unnecessarily puts the patient at risk for potential adverse events. The results of this study emphasize the importance of examining the patient's need for AST at each level of care especially when the identified risk factors are present.

## 1. Introduction

Acid suppressive therapy (AST), mainly proton pump inhibitors (PPIs) and histamine-2 receptor antagonists (H2RAs), refers to highly effective acid suppressants with decades of use with generally favorable outcomes in millions of patients worldwide. AST is indicated for a number of acid-related disorders, including treatment of upper gastrointestinal (GI) bleeding, gastroesophageal reflux disease (GERD), erosive gastritis or esophagitis, dyspepsia, reduction of GI ulcers, complications of patients taking nonsteroidal anti-inflammatory drugs (NSAIDs), and stress ulcer prophylaxis (SUP) in high-risk patients. Current evidence suggests that AST is commonly prescribed for SUP for hospitalized noncritically ill patients without an appropriate indication [1, 2]. AST is considered overutilised when prescribed without an appropriate indication. Moreover, patients may be left on AST “indefinitely” without appropriate indications when it is continued on discharge.

The untoward effects related to this practice are profound. Subjecting patients to unnecessary adverse drug reactions is a major concern. Increased risk of hospital-acquired pneumonia,* Clostridium difficile *infection, and drug-drug, drug-nutrient, and drug-test interactions have been linked to PPI therapy, although most data are from retrospective observational studies that may be subject to confounding and bias. Also, since AST may be started and continued even after discharge without justification, this practice surely adds to unnecessary healthcare costs.

Reasons behind unjustified AST prescribing habits are unclear [3, 4]. Recently there have been a number of studies focusing on these issues. By examining the prescribing patterns and inappropriate use of AST during hospitalization and at discharge from 2011 to 2013 at our community hospital, we sought to identify the risk factors associated with such practices.

## 2. Methods

In this retrospective observational study, we reviewed inpatient records of patients admitted to all medical and surgical services in both Intensive Care Unit (ICU) and non-ICU setting from January 2011 to December 2013 and looked at the trend of AST use during that time period.

### 2.1. Inclusion and Exclusion Criteria

 
*The inclusion criteria*
  1. Any adult patient admitted during January 2011 to December 2013
  2. All adult patients admitted to hospital between study periods who received at least one dose of any kind of AST
 
*The exclusion criteria *
  1. Patients less than 18 years of age
  2. Any patient with incomplete medical records
  3. Deceased patients

### 2.2. Sample Size

**Figure 1:**
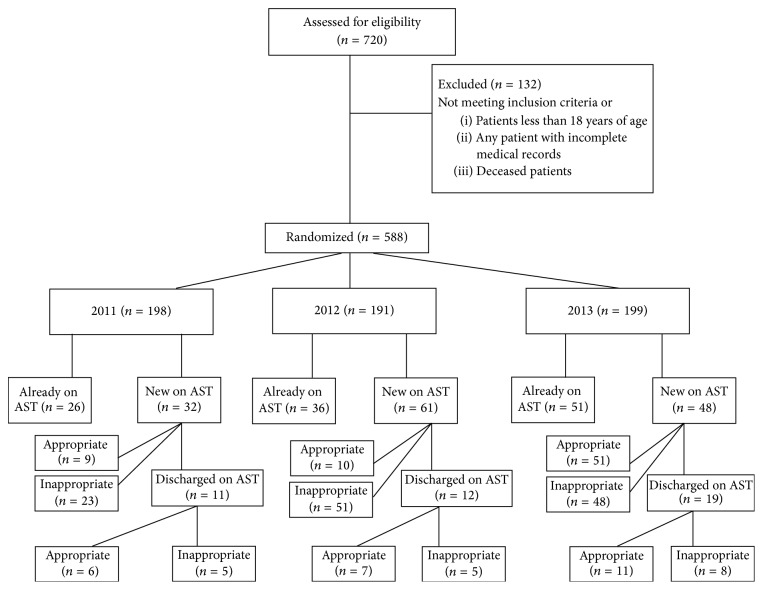
AST use during hospitalization from 2011 to 2013. AST: acid suppressive therapy.

Chart reviews were performed on 20 random patients per month from January 2011 to December 2013. Of the total 720 charts reviewed, 588 were included in the study after exclusion. Out of 588 included we sought out patients who were on AST and then classified those patients into two groups. Group one was already on AST, and the second group was newly started on AST during hospitalization. From here we then determined how many of those patients who were newly started on AST were for appropriate reasons (Figure 1).

### 2.3. Appropriateness of AST

Treatment with AST for non-SUP indications was considered appropriate, as supported by medical literature, if the patient had a specific indication or appropriate treatment purpose (e.g., GERD, PUD, dyspepsia, or acute or suspected GI bleeding). SUP was defined as acid suppressing medication given to prevent stress ulcer bleeding in the absence of current evidence of bleeding. Appropriate administration of SUP was derived from an internal guideline that is based on the ASHP guidelines. Prophylaxis was considered appropriate if a patient had one absolute indication [coagulopathy (defined as platelet count <50,000 mm^3^ or an international normalization ratio of >1.5, or a partial thromboplastin time >2 times the control value, or requiring mechanical ventilation for more than 48 h)] or 2 or more relative indications [sepsis, occult bleeding, use of high dose corticosteroids (>250 mg/d of hydrocortisone or the equivalent), recent use of NSAIDs for more than 3 months, renal failure (end-stage renal disease or kidney transplantation), liver failure (cirrhosis or liver transplantation), enteral feeding, and anticoagulant use].

Out of the patients started on AST inappropriately each year we determined if they were also discharged on AST.

We also looked at the variables associated with inappropriate use of AST including age, sex of patients, length of stay, medical versus surgical services, teaching versus nonteaching services/hospitalist, and resident/intern versus attending physician as different risk factors for inappropriate use of AST.

### 2.4. Statistical Analysis

A logistic regression model was used to examine risk factors associated with inappropriate AST use. Two-sample *t*-tests were used to compare age and length of stay by inappropriate AST use. Fisher's exact tests were used to compare inappropriate use by categorical variables (sex, number of handoffs (0 versus 1+), teaching versus nonteaching services, and admitting physician (attending versus resident versus hospitalist versus other)). Differences in percentages were assessed using the *v*2 test, and differences in means were assessed using Student's *t*-test. A *p* value of 0.05 or less was considered to be significant.

## 3. Results

**Figure 2:**
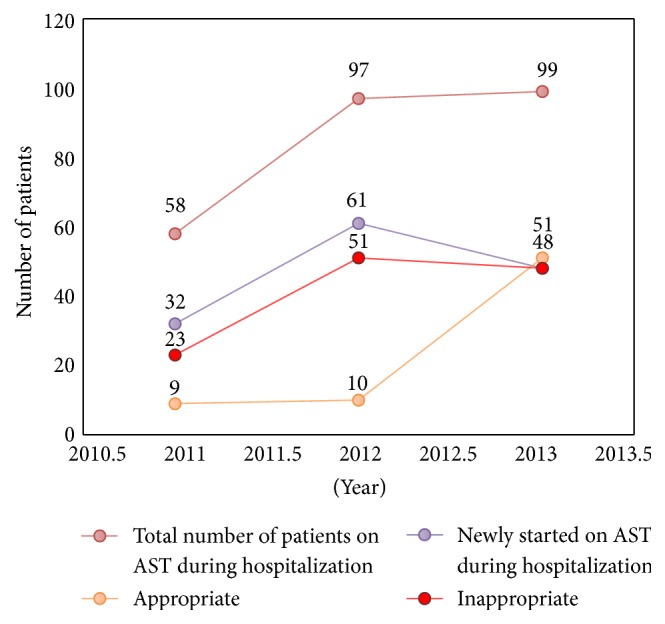
AST use at discharge during 2011–2013. AST: acid suppressive therapy.

**Figure 3:**
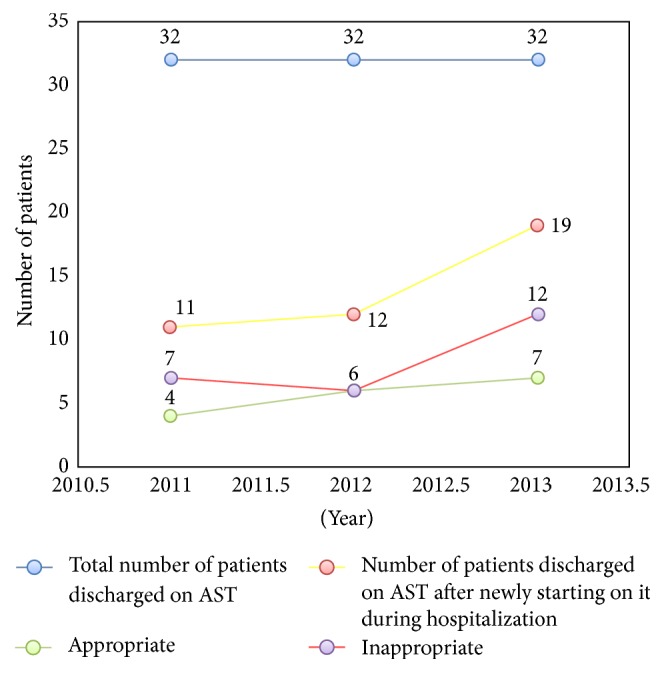
Consort diagram showing the AST prescribing pattern at a community hospital from January 2011 to December 2013. AST: acid suppressive therapy.

240 patient charts were reviewed each year. After excluding the newborn, children, and deceased and those who had missing data on admission, total patients for 2011, 2012, and 2013 were 198, 191, and 199, respectively. Number and trend of patients on AST and being appropriate cases or not through 2011–2013 are shown in Figures 2 and 3. The total number of patients admitted in 2011 was 198, 58 of whom were on AST (26 or 45% were already on AST and 32 or 55% were newly started on it). Among the 32 newly started on AST, 23 or 72% were inappropriate cases. Also, among the 32 newly started on AST, 11 or 34% were known to be discharged on AST, 5 or 45% of whom were known to be inappropriate cases. Six or 26% of the 23 inappropriately started on AST were known to be further discharged on it.

The total number of patients admitted in 2012 was 191, 97 of whom were on AST (36 or 37% were already on AST and 61 or 63% were newly started on it). Among the 61 newly started on AST, 51 or 84% were inappropriate cases. Also, among the 61 newly started on AST, 12 or 20% were known to be discharged on AST, 5 or 42% of whom were known to be inappropriate cases. Five or 10% of the 51 inappropriately started on AST were known to be further discharged on it.

The total number of patients admitted in 2013 was 199, 99 of whom were on AST (51 or 52% were already on AST and 48 or 48% were newly started on it). Among the 48 newly started on AST, 36 or 75% were inappropriate cases. Also, among the 48 newly started on AST, 19 or 40% were known to be discharged on it, 8 or 42% of whom were known to be inappropriate cases. 10 or 28% of the 36 inappropriately started on AST were known to be further discharged on it (Figure 3).

In total, 21 or 19% of the 110 inappropriately started on AST were found to be further discharged on AST over the three years.

### 3.1. Indications of AST

*At Admission*. Except for AST started with no indication (48/34.04%), GI prophylaxis was the most commonly used indication to start AST (37/26.24%), followed by GI bleed (12/8.51%), GERD (5/3.55%), and abdominal pain (4/2.84%). However, GI prophylaxis was regarded as totally inappropriate though it was most commonly used. GI bleed and GERD were considered as appropriate indications. For abdominal pain, 3 cases were considered as inappropriate and 1 case was considered as appropriate (Table 1).

*At Discharge*. Except for no indication (18/42.86%), the most commonly used indication for being newly started and then discharged on AST was GI bleed (6/14.29%), followed by GERD (4/9.52%). Both were considered as appropriate (Table 2).

### 3.2. Risk Factors

The results (Table 3) demonstrate that age was significantly associated with outcome (*p* value = 0.0306). The results of the logistic regression indicated that younger age (OR = 0.962, *p* = 0.0029) is associated with inappropriate use; for each year increase in age, the odds of inappropriate use decrease by (1–0.962) = 3.8%. Number of handoffs (0 versus 1+) (*p* value = 0.0162) was also significantly associated with inappropriate use. Having at least one handoff (OR = 9.103, *p* = 0.0355) is significantly associated with inappropriate use. The odds of inappropriate use for patients with at least one handoff between services are about 9-fold those of patients without any handoffs between services.

We also examined the effects of these factors on the inappropriate use of AST at discharge. Being female (OR = 6.050, *p* = 0.0313) is associated with an increase of inappropriate AST at discharge. The odds of inappropriate AST at discharge for female patients are about 6-fold those of male patients. Longer LOS (OR = 1.186, *p* = 0.0608) was marginally associated with inappropriate AST at discharge. In other words, for each day increase in length of stay, the odds of inappropriate AST at discharge increase by (1.186–1) = 19%.

## 4. Discussion

Today's healthcare system is going through a new phase with much focus on cost-effectiveness, patient safety, and quality of care. There have been many publications regarding inappropriate use of AST during hospitalization and for outpatients. However, this trend seems to be continuing. The most frequently seen indication at the time of admission and at discharge is usually antiplatelet therapy. During hospitalization, it is prophylaxis for stress ulcer in patients at low risk [5]. As shown by our study, more than 70% of newly prescribed AST each year during hospitalization was inappropriate, with similar rates each year over the study period from January 2011 to December 2013 (71.88 in 2011, 85.25% in 2012, and 78.72% in 2013) at our community hospital. This percentage was greater than what has been reported by others (in the range of 27%–71%) [3, 4]. Of the patients included in the study period, 34.04% received AST without an indication, followed by GI prophylaxis (26.24%) and GI bleeding (8.51%) as major indications to start AST during hospitalization both in ICU and in non-ICU settings. And these practices were not any different among different medical and surgical services, among resident/intern versus attending physicians, and among teaching versus nonteaching services. Once AST is started inappropriately, there is a high likelihood that patients will be discharged on it and will continue on it without any indication [4–6]. This was also supported by our study as 19% of the patients inappropriately started on AST got discharged inappropriately on it.

Our results agree with previous reports on the overuse of AST in hospitalized patients. Nardino et al. [3] reported the overuse of AST in a large community hospital in the United States where 54% of hospitalized patients received AST, 65% of whom were inappropriate cases. Parente et al. [4] reported that, in hospitalized patients receiving AST, 68% of prescriptions were inappropriate, most of which were for SUP in low-risk patients. Similar results were seen in Zink et al. [6], where 60% of patients were started on AST without a clear or valid indication. Furthermore, Yap and Chan [7] studied the prescribing patterns of Singapore hospitals and found that acid suppression medications were the second most commonly prescribed drugs in the medical wards and emergency services. As mentioned earlier, in our study, a major proportion of unnecessary AST was used without indication followed by GI prophylaxis. Although the indications for AST for the treatment of acid-related diseases and the prevention of gastric mucosal damage in an ICU setting have been well defined in the medical literature, in recent years the practice of SUP has become increasingly common in general medicine patients, with little to no evidence to support it. We assessed the appropriateness of SUP based on an internal guideline that is derived from the only published guidelines addressing SUP, the ASHP guidelines [2]. Since the guidelines only endorse SUP for selected ICU patients, there is an urgent need for SUP guidelines for patients outside the ICU. Whether any non-ICU patients should receive SUP needs to be determined.

Studies done in the past tried to identify associated and predictive factors for the inappropriate prescribing of AST in an attempt to guide future practice. A review of the published data shows conflicting endpoints. Our results suggest that* younger age* is a clear variable for AST misuse. This is in contradiction to previous studies [8, 9]. These results are very surprising as one would consider older patients at higher risks of AST misuse due to their multiple medical problems and increased number of prescribed medications.

Another interesting finding is association of* female sex* with inappropriate use of AST. One study has shown that female gender was independently associated with inappropriate community prescribing among elderly UK primary care patients [10]. Another study revealed female gender to be strongly predictive of inappropriate IV PPI prescribing [11]. Recent data found no difference between appropriate and inappropriate use of intravenous PPIs in non-ICU male and female patients [12]. An explanation for these results was not offered.

Another variable studied was the* number of handoffs* between different services which was found to be statistically significant as a risk factor for inappropriate AST use. We believed this represented a higher level of care that patients were initially admitted to. Therefore, it was likely that physicians “overprescribed” AST based on how sick patients were perceived to be even if no criteria were met. Also, in our experience, AST is often overlooked during medication reconciliation at the time of transfer between two services, leading to continuation of therapy without any significant indication. This is likely related to “clinical inertia” and at times this led to patients being discharged on AST when there was never a clear indication.

Similarly,* length of hospital stay* was noted to be another factor marginally impacting AST use. For each day increase in length of stay, the odds of inappropriate AST at discharge increased by (1.186–1) = 19%. Issa et al. found similar results as well [8].

Our study did not find any association between teaching versus nonteaching services, medicine versus surgical services, and attending physician versus resident admissions as a risk factor for inappropriate AST use. These findings are in contrast to previously published studies [11] where admission to the surgical ward seems to be a risk factor for inappropriate SUP use. A more recent prospective trial assessed intravenous PPIs in ICU and non-ICU patients and reported no difference in appropriateness with regard to different specialties or even various departments [13]. Also, in contrast to previous study, we could not find any difference in prescribing pattern between residents and attending physicians [14], as well as teaching versus hospitalist services.

In our hospital, 65% of the used AST was shown to be a PPI. This is compatible with the current practice trends and although randomized data is still lacking with regard to PPIs in reducing gastrointestinal bleeding from stress ulceration (as compared to placebo), these agents are currently the most common prescribed ones for this condition [14–16].

Although AST is often viewed as safe, it is associated with increased colonization of the upper GI tract with potentially pathogenic organisms, which was found to increase the risk of hospital-acquired pneumonia [17]. In addition, gastric acid is an important defense against the acquisition of* Clostridium difficile *spores and, by increasing gastric pH when using AST, the risk of* Clostridium difficile *infection will increase [18, 19]. Furthermore, AST has the potential for drug-drug, drug-nutrient, and drug-test interactions through a variety of mechanisms, as well as having agent specific side effects [2]. The practice of prescribing SUP in non-ICU hospitalized patients has substantial financial ramifications for both patients and hospitals. If we are not careful in our prescribing pattern, we can start a vicious cycle, which increases the cost burden on health system and puts patients at higher risks of side effects and adverse drug reactions. One retrospective cohort study conducted in a single US academic hospital setting found inpatient costs for SUP in the non-ICU setting to be $44,000 annually, coupled with nearly $68,000 in outpatient pharmacy costs when the PPIs were reflexively continued upon hospital discharge, for a combined estimated expenditure of nearly $112,000 annually, which could have easily been prevented through institution of and adherence to proper guidelines for SUP [20].

In order to reduce the risk of initiating and continuing inappropriate AST, we suggest the following interventions: (1) All house staff should be educated about this problem with special emphasis on evaluating and reevaluating the need for AST at every level of care. (2) Every healthcare system should have strict guidelines on indications when AST is appropriate. (3) The pharmacy should have a pivotal role in making sure these guidelines are adhered to. Liberman and Whelan [21] were able to reduce the rate of inappropriate SUP significantly by sparing one out of every 3 patients an inappropriate medication by conducting a low-cost educational intervention based on the principles of practice-based learning and improvement. Also pharmacy intervention has been shown to reduce inappropriate AST use [22–24].

There are a few* limitations* to the results of our study.

First of all, it was conducted in a single center; although it was a large hospital, one may suggest bias due to a small group of physicians working within one specific healthcare setting. Second, data was retrieved by retrospective chart review. Third, we recognize that practices at a community-based academic medical center has several features that may not apply to other hospitals. For instance, most initial decision-making and order entry are done by an intern or a resident. Fourth, the line between appropriate and inappropriate uses of PPIs can often be blurry at times and there are no specific guidelines to call upon.

*Strengths* of study include the following: (a) Number of patients was adequate (*n* = 588) to see the trend over study period. (b) Our database is electronic, and, therefore, the possibility of missing patients or cases is highly unlikely. For scanned paper charts, we excluded all patients with any missing data. (c) Both ICU and non-ICU adult patients were selected because we wanted to eliminate the bias of patient being sicker while in ICU. Patients were monitored and followed up during their hospital stay until discharge or switching off. Although our results might not be generalizable to other nonacademic centers, our practices are reflective of other similar hospitals across the region.

In our study, there was a high frequency of unnecessary use of AST in hospitalized patients with inappropriate continuation after discharge. Unnecessary AST can increase healthcare cost and adverse drug related events. The results of our study highlight the need for interventions, including implementation of institutional protocols and prescriber education regarding use of AST during hospitalization and at discharge.

## Figures and Tables

**Table 1:** Indications to start AST.

	Indication	Percent	Appropriate or not
1	No indication	34.04	No
2	GI prophylaxis	26.24	No
3	GI bleed	8.51	Yes
4	GERD	3.54	Yes
5	Abd. pain	2.83	3 no/1 yes
6	ASA/Plavix	1.41	Yes
7	Fracture	1.41	No
8	Gastritis	1.42	Yes
9	2 anticoag.	0.70	Yes
10	Others	19.9	No

**Table 2:** Indications for discharge on AST.

	Indication	Percent	Appropriate or not
1	No indication	42.86	No
2	GI bleed	14.28	Yes
3	GERD	9.52	Yes
4	GI prophylaxis	7.14	No
5	Gastritis	4.76	Yes
6	Abd. pain	2.40	Yes
7	Anticaogulants	2.40	Yes
8	As needed?	2.38	No
9	Chronic steroids	2.38	Yes
10	Others	11.88	Yes

**Table 3:** Analysis of maximum likelihood estimates.

Parameter		Standard error	Wald Chi-square	OR	95% CI	*p* value
Sex	Female	0.8362	4.6347	6.050	1.175–31.156	**0.0313**
Age		0.0130	8.8918	0.962	0.938–0.987	**0.0029**
Number of handoffs between services	≥1	1.0505	4.4205	9.103	1.162–71.340	**0.0355**
Length of stay		0.0911	3.5155	1.186	0.992–1.418	0.0608
